# Concussion Disrupts Normal Brain White Matter Microstructural Symmetry

**DOI:** 10.3389/fneur.2020.548220

**Published:** 2020-11-12

**Authors:** Jun Maruta, Jacob M. Mallott, Gary Sulioti, Jamshid Ghajar, Eva M. Palacios, Pratik Mukherjee

**Affiliations:** ^1^Department of Neurology, Icahn School of Medicine at Mount Sinai, New York, NY, United States; ^2^Department of Rehabilitation and Human Performance, Icahn School of Medicine at Mount Sinai, New York, NY, United States; ^3^Brain Trauma Foundation, New York, NY, United States; ^4^Department Radiology and Biomedical Imaging, University of California, San Francisco, San Francisco, CA, United States; ^5^Duke University School of Medicine, Durham, NC, United States; ^6^Department of Neurosurgery, Stanford University School of Medicine, Stanford, CA, United States; ^7^Department of Bioengineering and Therapeutic Sciences, University of California, San Francisco, San Francisco, CA, United States

**Keywords:** acute concussion, bilateral homolog, diffusion tensor imaging (DTI), mild traumatic brain injury (mTBI), magnetic resonance imaging (MRI)

## Abstract

Injuries and illnesses can alter the normal bilateral symmetry of the brain, and determining the extent of this disruption may be useful in characterizing the pathology. One way of quantifying brain symmetry is in terms of bilateral correlation of diffusion tensor metrics between homologous white matter tracts. With this approach, we hypothesized that the brains of patients with a concussion are more asymmetrical than those of healthy individuals without a history of a concussion. We scanned the brains of 35 normal individuals and 15 emergency department patients with a recent concussion. Fractional anisotropy (FA), mean diffusivity (MD), axial diffusivity (AD), and radial diffusivity (RD) were determined for regions of interest (ROI) defined by a standard white-matter atlas that included 21 bilateral ROIs. For each ROI pair, bilateral correlation coefficients were calculated and compared between the two subject groups. A symmetry index, defined as the ratio between the difference and the sum of bilateral measures, was also calculated for each ROI pair and compared between the groups. We found that in normal subjects, the extent of symmetry varied among regions and individuals, and at least subtle forms of structural lateralization were common across regions. In patients, higher asymmetry was found overall as well as in the corticospinal tract specifically. Results indicate that a concussion can manifest in brain asymmetry that deviates from a normal state. The clinical utility of characterizing post-concussion pathology as abnormal brain asymmetry merits further exploration.

## Introduction

Although the functional and anatomical lateralization of the brain is well recognized, what is more obvious, and thus can easily be taken for granted, is its normal structural symmetry. There may be various ways to quantify bilateral symmetry of the brain, but one approach is to do so for the microstructure of white matter tracts in terms of correlation between the metrics of magnetic resonance (MR) diffusion imaging. As is known, correlations of diffusion metrics between homologous pairs of tracts are generally high in healthy individuals (1–3). This verification supports an assertion that bilateral asymmetry reflects disruption of a normal state when found in patients with injuries or illnesses and that determining the extent of this disruption is useful in characterizing the pathology (2, 4, 5). Still, it needs to be noted that there are normal asymmetries as well as symmetrical abnormalities. To examine *abnormal* brain asymmetry, at least three considerations are needed: (1) the extent and similarity of the impact of an injury or illness on bilateral regions vary; (2) the degrees of normal symmetry vary among regions (1); and (3) no two injuries or illness progressions are alike (2).

Here, using MR diffusion tensor imaging (DTI) we studied white matter microstructural symmetry of adults with and without a concussion, with the hypothesis that the brains of patients with a concussion are more asymmetrical. We investigated abnormal brain asymmetry by: (1)′ examining whether any white matter tract is potentially more vulnerable to concussion than others; (2)′ benchmarking the normal extent of symmetry between homologous pairs of white matter tracts; and (3)′ examining deviations from these benchmarks at an individual level. We also controlled for potential developmental variables by examining only the data from subjects 18 years or older.

This report constitutes a secondary substudy of a larger study related to concussion with a focus on a normative characterization of eye movement performance in a variety of cohorts (6, 7) and is cross-sectional case-control in design. MR images were collected from predefined groups of subjects. Some imaging results not overlapping with the present report, comparing concussed young athletes and patients from the emergency department (ED), have been published (8). Here we focused on ED patients because a substantial number of adults were included in the sample and their demographic characteristics agreed well with those of the control pool.

## Methods

### Subjects

The protocols for subject enrollment and assessment were approved by the Institutional Review Board of Weill Cornell Medical College (Approval number: 1201012120). Patients from the ED with concussion within the past 2 weeks and healthy control subjects with no history of head injury, aged 7 years or older, were recruited for the imaging study. Control subjects were recruited through flyers posted at colleges, office buildings, community centers, and other facilities in the New York City area. A concussion was defined as an event of blunt impact on the head, with loss of consciousness (LOC), post-traumatic amnesia (PTA), or at least one of the following symptoms: dizziness, nausea, headaches, balance problems, blurred or double vision, or feeling dazed/confused. Although for the purpose of this research we did not rely on formal medical diagnosis of concussion, this definition is consistent with the guidance of the American Academy of Neurology (9). Prior to data collection, written informed consent by adult subjects, or legal guardians of minor subjects with the minors' assent, was obtained in accordance with the Declaration of Helsinki. Subjects' symptoms and cognitive performance were assessed with an extensive battery of tests as reported elsewhere (7).

As stated in Introduction, only the data from subjects 18 years and older were analyzed for this substudy. These subjects were required to have a high school diploma or GED, or for 18-year olds, set to graduate high school on time. Exclusion criteria as per the parent study were a prior history of eye disease, neurological/psychiatric conditions, substance abuse, or contraindications for MR imaging. For ED patients with concussion, additional exclusion criteria were acute intoxication at the time of the concussion and LOC or PTA for more than 24 hours. Of the total of 42 patients, 15 were 18 years or older. Recruited patients were MR-scanned as soon as scheduling allowed. The scan took place with a mean (SD) of 10.5 (2.8) days following the injury. A total of 38 control subjects were MR-scanned, of whom 35 were 18 years or older. A comparison of the groups' demographic characteristics is reported in Results.

### Magnetic Resonance Imaging

The methods for MR image acquisition, quality inspection, and processing were described previously (8). Briefly, on a 3T Siemens Trio scanner, whole-brain diffusion imaging with 128 × 128 × 60 cubic voxels of 2 mm dimensions was conducted using an echo-planar imaging sequence (TE = 85 ms, TR = 7500 ms) with one *b* = 0 s/mm^2^ scan and *b* = 1,000 s/mm^2^ in 64 diffusion directions. Images were processed with tools within the Functional MRI of the Brain (FMRIB) Software Library (10). They were corrected for eddy currents and subject motion and registered to the *b* = 0 s/mm^2^ volume using the FMRIB's Linear Image Registration Tool (11). Image volumes were checked for excessive subject movement between diffusion weighted images and were accepted for mean and median movement being <2 mm. Non-brain voxels were excluded using the Brain Extraction tool (12). Using the diffusion-weighted data, a diffusion tensor model was generated using DTIFIT, from which fractional anisotropy (FA), mean diffusivity (MD), radial diffusivity (RD), and axial diffusivity (AD) were determined at each voxel. These interdependent metrics together characterize local water diffusion properties.

Tract-Based Spatial Statistics (TBSS) were used to perform non-linear registration on the FA volumes to the FMRIB58_FA standard-space, constructed from the average of 58 FA images of healthy adult subjects (13, 14). After brain volumes were registered into the common space, a mean FA skeleton was generated using a threshold of FA ≥ 0.2 to limit the analysis to white matter voxels. TBSS alignment and white-matter skeleton generation were performed separately for each subject group. Masks were applied corresponding to the 48 white matter tracts labeled in the Johns Hopkins University white-matter atlas (15). The regions of interest (ROI) included 21 pairs of bilateral tracts: anterior corona radiata; anterior limb of the internal capsule; cingulum-cingulate gyrus; cingulum-hippocampus; cerebral peduncle; corticospinal tract; external capsule; fornix/stria terminalis; inferior cerebellar peduncle; medial lemniscus; posterior corona radiata; posterior limb of the internal capsule; posterior thalamic radiation; retrolenticular part of the internal capsule; superior cerebellar peduncle; superior corona radiata; superior fronto-occipital fasciculus; superior longitudinal fasciculus; sagittal stratum; tapetum; and uncinate fasciculus. Note that in this atlas, the middle cerebellar peduncle was not defined bilaterally. For each subject, within-ROI mean values of the four DTI metrics were determined.

### Statistics

Group-wise matching in age was tested with an F-test and a two-sample *t*-test. Matching in sex was tested with a chi-square test. The alpha level was set to 0.05.

To identify particular white matter tracts as potentially vulnerable to concussive impacts, we compared DTI metric values of each of the 48 ROIs between the two groups using a two-sample *t*-test without assuming equal variances, where data from all subjects were entered for each ROI. A statistically significant difference between the means was taken as an indication of propensity for concussive injury. The alpha level was corrected for multiple comparison using Holm's method considering the number of ROIs (stepping down from α/48 = 0.00104, α/47 = 0.00106, and so on, until no further null hypothesis could be rejected) (16). We inspected group differences in variances in DTI metrics using an F-test, where data from all subjects were entered for each ROI. Furthermore, we examined whether there was an overall trend in mean or variance differences across ROIs using a paired *t*-test with pairing by ROI, where the within-group mean or variance of 48 ROIs were entered.

For each of 21 ROI pairs for each DTI metric, Spearman's correlation coefficients ρ between the two sides of the brain were calculated for the two groups. A one-tailed paired *t*-test, paired between groups for each ROI, was used to test the hypothesis that the mean bilateral correlation was reduced in patients compared to controls. To test whether specific ROIs could be identified as having lower correlations in patients, the ρ values were compared using Fisher's method (17). Based on the hypothesis that correlations would be reduced in patients, these tests were also one-tailed. The alpha level was corrected for multiple comparison using Holm's method.

Differences between patients and controls in bilateral symmetry of DTI metrics were also examined using an index defined as the ratio between the difference (right minus left) and the sum of bilateral measures within individual. By definition, the value of this symmetry index ranges from −1 to 1, with 0 indicating perfect symmetry. Distributions of symmetry indices were visualized with box plots. The bottom and top of each box indicate the first (Q1) and third (Q3) quartiles, and the middle line or symbol the median. The whiskers indicate the minimum and maximum of all the data excluding outliers whose values were larger than Q3 + w · (Q3 – Q1) or smaller than Q1 – w · (Q3 – Q1), where w = 1.5. We expected that the index values of some patients would fall outside the normal spread. Specifically, we hypothesized that there would be ROIs in which values of the symmetry index were more variable among patients than controls. This hypothesis was tested using a one-tailed F-test. The alpha level was corrected for multiple comparison using Holm's method considering that there were 21 bilateral ROI pairs.

Although normal white matter microstructural asymmetry is already recognized (1), we sought to document it as well using the symmetry index. We applied a two-tailed one-sample *t*-test to the control sample to identify tracts whose symmetry index values deviated from zero. Again, the alpha level was corrected for multiple comparison using Holm's method.

Finally, it was hypothesized that, with abnormal values of symmetry indices expected among patients, the *magnitudes* of the symmetry indices across ROIs would be overall larger in patients than controls. This hypothesis was tested by taking within-individual averages of the absolute values of symmetry indices across ROIs and applying a one-tailed *t*-test between the two groups.

## Results

The mean (SD) ages of the ED patient and control groups were 35.5 (12.7) and 40.7 (15.4) years old, respectively. The patient group was 60% female while the control group was 66% female. The two groups did not differ significantly in terms of distributions in age [F_(34, 14)_ = 1.41, *p* = 0.50; |*t*(48)| = 1.13, *p* = 0.26] or sex [χ^2^(1) = 0.15, *p* = 0.70].

Group-wise comparisons of FA values of 48 ROIs yielded statistical differences after correction for multiple comparison in the middle cerebellar peduncle, right corticospinal tract, splenium of the corpus callosum, right posterior limb of the internal capsule, and right superior fronto-occipital fasciculus (Table 1). Comparisons of MD values yielded differences in the bilateral superior fronto-occipital fasciculus, genu and body of the corpus callosum, left anterior corona radiata, right anterior limb of the internal capsule, and right posterior limb of the internal capsule. Comparisons of RD values yielded differences in the right superior fronto-occipital fasciculus, right posterior limb of the internal capsule, middle cerebellar peduncle, genu and splenium of the corpus callosum, left anterior corona radiata, and left external capsule. Finally, comparisons of AD values yielded differences in the left superior fronto-occipital fasciculus.

**Table 1:** White matter tracts that yielded a statistically significant difference between patients with concussion and control subjects in each DTI metric.

	**Patient mean**	**Control mean**	**|*t*|**	**df**	***p***
**FA**					
Middle cerebellar peduncle	0.563	0.598	5.92	35.7	<0.0001
Cortico-spinal tract, R	0.616	0.579	4.30	28.6	0.0002
Splenium of corpus callosum	0.819	0.801	3.87	45.4	0.0003
Posterior limb of internal capsule, R	0.701	0.678	3.72	33.1	0.0007
Superior fronto-occipital fasciculus, R	0.548	0.507	3.79	27.0	0.0008
**MD**					
Superior fronto-occipital fasciculus, L	6.31 × 10^−4^	6.68 × 10^−4^	4.57	36.3	<0.0001
Genu of corpus callosum	6.92 × 10^−4^	7.25 × 10^−4^	4.36	45.5	<0.0001
Anterior corona radiata, L	7.13 × 10^−4^	7.46 × 10^−4^	4.29	40.9	0.0001
Superior fronto-occipital fasciculus, R	6.30 × 10^−4^	6.69 × 10^−4^	4.39	32.0	0.0001
Anterior limb of internal capsule, R	6.85 × 10^−4^	7.10 × 10^−4^	4.15	40.0	0.0002
Posterior limb of internal capsule, R	6.95 × 10^−4^	7.18 × 10^−4^	4.24	31.9	0.0002
Body of corpus callosum	7.73 × 10^−4^	8.03 × 10^−4^	3.68	43.1	0.0006
**RD**					
Superior fronto-occipital fasciculus, R	4.14 × 10^−4^	4.60 × 10^−4^	6.03	46.5	<0.0001
Posterior limb of internal capsule, R	3.56 × 10^−4^	3.86 × 10^−4^	4.73	34.7	<0.0001
Middle cerebellar peduncle	4.68 × 10^−4^	4.27 × 10^−4^	4.65	22.8	0.0001
Genu of corpus callosum	2.67 × 10^−4^	3.09 × 10^−4^	4.16	40.2	0.0002
Anterior limb of internal capsule, R	4.05 × 10^−4^	4.29 × 10^−4^	4.08	44.5	0.0002
Splenium of corpus callosum	2.43 × 10^−4^	2.72 × 10^−4^	4.02	42.3	0.0002
Anterior corona radiata, L	4.93 × 10^−4^	5.29 × 10^−4^	3.72	36.7	0.0007
External capsule, L	5.28 × 10^−4^	5.52 × 10^−4^	3.49	43.9	0.0011
**AD**					
Superior fronto-occipital fasciculus, L	1.03 × 10^−3^	1.08 × 10^−3^	4.29	27.6	0.0002

*Group means and the results of two-sample t-tests are shown. Statistical significance was determined by correction of α-levels for multiple comparison. L, left; R, right; df, degrees of freedom*.

**Figure 1:**
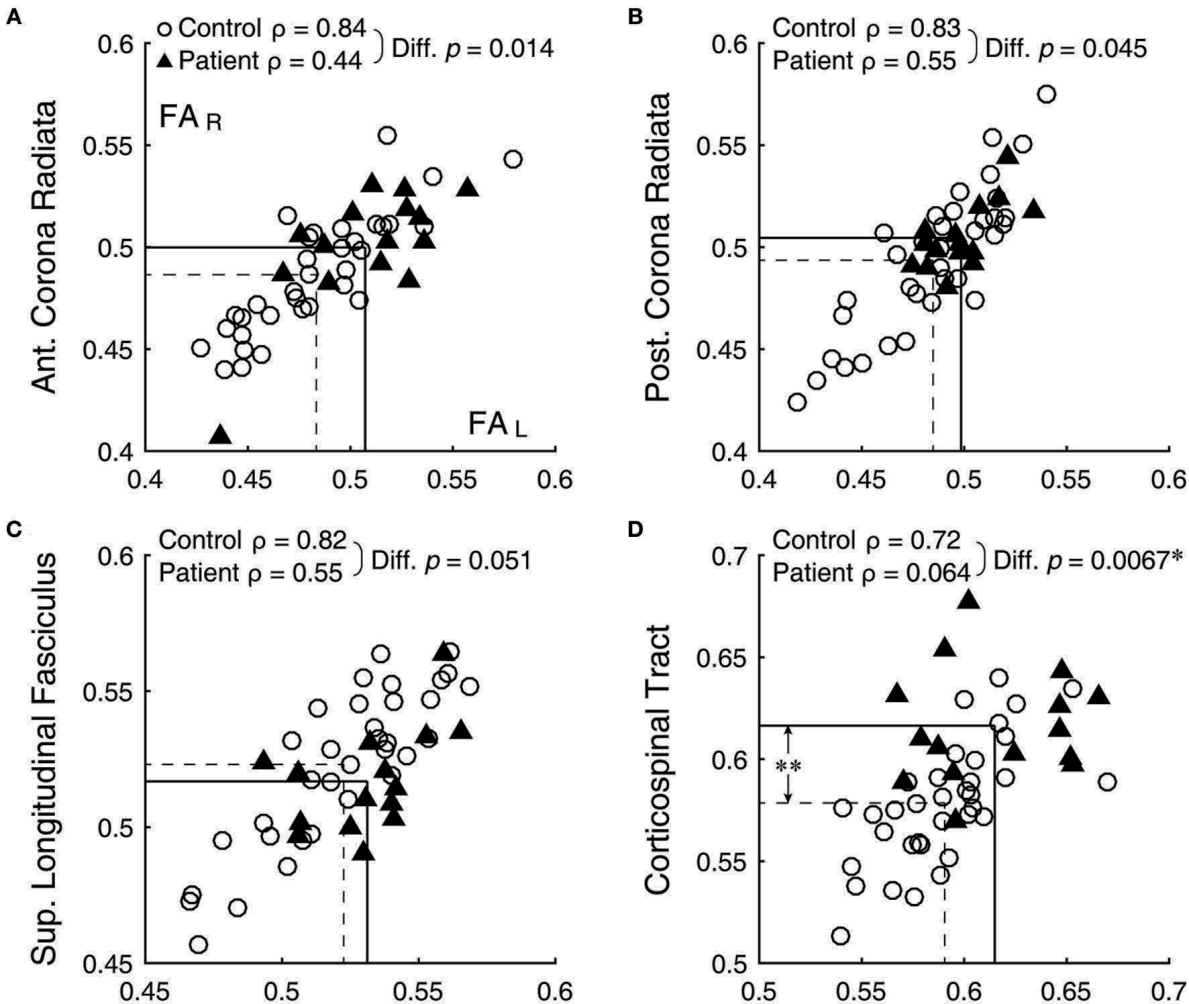
Bilateral correlation of FA values. **(A)** anterior corona radiata, **(B)** posterior corona radiata, **(C)** superior longitudinal fasciculus, and **(D)** corticospinal tract. Open circles and filled triangles indicate control and patient subjects, respectively, with the left and right FA values represented by the abscissa and ordinate, respectively. Dashed lines indicate means of the control group and solid lines those of the patient group. *Significant difference (with correction for multiple comparison) between the ρ-values. **Significant difference (with correction for multiple comparison) between the means (see Table 1).

The above listed tracts were identified as possibly vulnerable to concussion. Even so, across ROIs, averaged FA values tended to be larger in the patient group [|*t*(47)| = 6.15, *p* < 0.0001], and MD, RD, and AD values smaller in the patient group [MD: |*t*(47)| = 7.56, *p* < 0.0001; RD: |*t*(47)| = 6.87, *p* < 0.0001; AD: |*t*(47)| = 4.73, *p* < 0.0001], substantiating diffuse effects of concussion. The statistically significant group differences found for the middle cerebellar peduncle in FA and RD values (Table 1) opposed these trends, however. In addition to the centrality measure, for each DTI metric, some ROIs indicated group differences in variances at the alpha level of 0.05. Although only the variances of MD values in the left cerebral peduncle showed a statistically significant group difference [*p* < 0.0001, F_(34, 14)_ = 8.88] when the alpha level was corrected for the number of ROIs, a general difference in the shapes of distributions could be noted. Specifically, the variances of FA values were significantly different between the two groups paired for ROIs [|*t*(47)| = 3.27, *p* = 0.0020], lower in the patient group. Tighter clustering of FA values are visualized in Figure 1 for 2 × 4 = 8 ROIs. There was no statistical group difference across ROIs in variances for MD, RD, or AD.

Bilateral correlation for the control group varied widely across ROIs, but ranged similarly among the DTI metrics: 0.30 ≤ ρ ≤ 0.89 for FA, 0.33 ≤ ρ ≤ 0.89 for MD, 0.19 ≤ ρ ≤ 0.89 for RD, and 0.14 ≤ ρ ≤ 0.84 for AD. Across the metrics, the anterior corona radiata, posterior corona radiata, and superior longitudinal fasciculus were among the consistently better-correlated ROIs, with ρ-values 0.75 or above (for example, Figures 1A–C, open circles). On the other hand, the uncinated fasciculus, fornix/stria terminalis, retrolenticular part of the internal capsule, and superior fronto-occipital fasciculus were among the consistently less-correlated ROIs, with ρ-values 0.70 or below.

Against the normative benchmark that bilateral ROIs have variable but mostly high ρ-values, in all DTI metrics but AD, ρ-values were significantly lower in the patient group than the control group [FA: *t*(20) = 2.40, *p* = 0.013; MD: *t*(20) = 3.08, *p* = 0.003; RD: *t*(20) = 2.67, *p* = 0.007; AD: *t*(20) = 0.01, *p* = 0.49]. Thus, bilateral symmetry was generally disrupted in patients as a group. Comparison of ρ-values associated with each bilateral ROI for the patient and control groups, after correcting the alpha level for multiple comparison, specifically identified the corticospinal tract as having a statistically significant reduction in the patient group (FA: *z* = 2.47, *p* = 0.007, Figure 1D; RD: *z* = 3.25, *p* < 0.001). Note that the corticospinal tract was also among the tracts with a statistically significant difference between the two subject groups (Table 1, larger FA in patients). Thus, this tract stood out as possibly generally vulnerable as well as variably sensitive to concussion.

**Figure 2:**
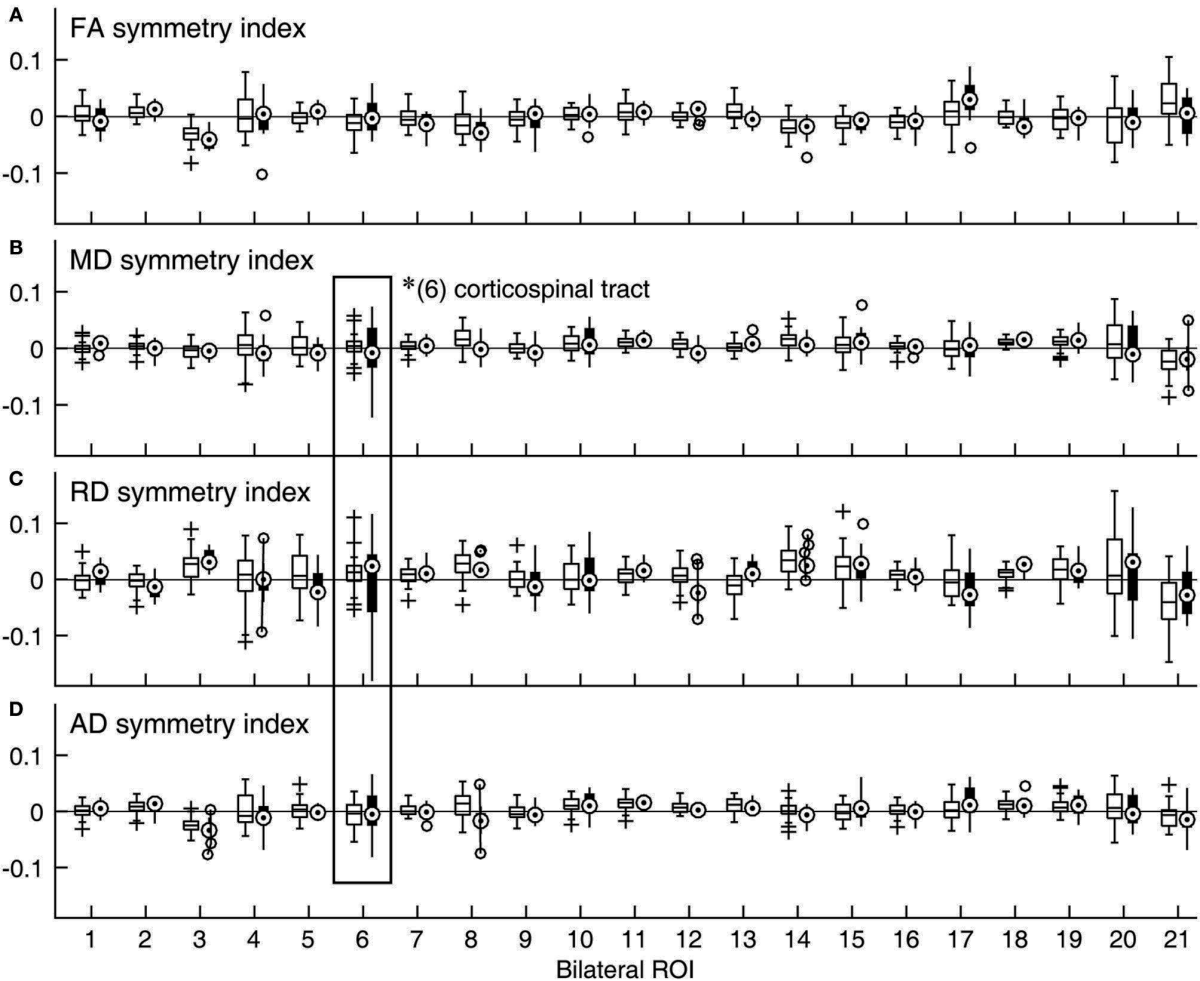
Distributions of symmetry indices across ROIs and DTI metrics expressed as boxplots. **(A)** FA, **(B)** MD, **(C)** RD, and **(D)** AD. A symmetry index value of zero indicates bilaterally equal DTI metric values (horizontal lines). For a given ROI, the data for the control group are shown with an open box on the right side, while those for the patient group are shown with a filled box on the left side. *Significant group difference (with correction for multiple comparison). (1) anterior corona radiata; (2) anterior limb of internal capsule; (3) cingulum-cingulate gyrus; (4) cingulum-hippocampus; (5) cerebral peduncle; (6) corticospinal tract; (7) external capsule; (8) fornix/stria terminalis; (9) inferior cerebellar peduncle; (10) medial lemniscus; (11) posterior corona radiata; (12) posterior limb of internal capsule; (13) posterior thalamic radiation; (14) retrolenticular part of internal capsule; (15) superior cerebellar peduncle; (16) superior corona radiata; (17) superior fronto-occipital fasciculus; (18) superior longitudinal fasciculus; (19) sagittal stratum; (20) tapetum; (21) uncinate fasciculus.

That normal variations in bilateral symmetry differed among ROIs was also characterized by the heterogeneity in the inter-individual variability of symmetry index values for the control group (Figure 2, open boxplots). The cingulum-hippocampus (ROI 4), tapetum (ROI 20), and uncinate fasciculus (ROI 21) were among those with the largest inter-individual variability across the four DTI metrics, while the anterior limb of the internal capsule (ROI 2), superior longitudinal fasciculus (ROI 18), and superior corona radiata (ROI 16) were among those with the smallest. The patient group also showed differences in the inter-individual variability of symmetry index values. However, between-group comparisons indicated that the variance in symmetry was larger in patients in the corticospinal tract yet again, as indicated in Figures 2B–D by longer whiskers for ROI 6, marked by a rectangle [MD: F_(34, 14)_ = 0.17, *p* < 0.0001; RD: F_(34, 14)_ = 0.14, *p* < 0.0001; AD: F_(34, 14)_ = 0.29, *p* < 0.0016].

Figure 2 also suggested that the distributions of symmetry index values often veered from zero. Indeed, in the majority of the bilaterally paired tracts of control subjects, a statistically significant deviation from zero was found for one or more DTI metrics (Table 2). Thus, at least subtle forms of structural lateralization were common in normal brains.

**Table 2:** White matter tracts with statistically significant asymmetry among normal individuals.

	**Mean**	**SD**	**|*t*(34)|**	***p***
**FA**				
Cingulum-cingulate gyrus	−0.031	0.018	10.20	<0.0001
Retrolenticular part of internal capsule	−0.019	0.017	6.54	<0.0001
Superior cerebellar peduncle	−0.012	0.016	4.38	0.0001
Uncinate fasciculus	0.029	0.039	4.34	0.0001
Superior corona radiata	−0.010	0.013	4.21	0.0002
Anterior limb of internal capsule	0.008	0.013	3.56	0.0011
Posterior thalamic radiation	0.009	0.017	3.29	0.0023
**MD**				
Superior longitudinal fasciculus	0.011	0.007	9.05	<0.0001
Posterior corona radiata	0.012	0.010	6.87	<0.0001
Retrolenticular part of internal capsule	0.015	0.015	5.69	<0.0001
Fornix/stria terminalis	0.017	0.018	5.64	<0.0001
Uncinate fasciculus	−0.023	0.025	5.33	<0.0001
Sagittal stratum	0.011	0.013	5.01	<0.0001
Posterior limb of internal capsule	0.007	0.011	4.12	0.0002
Superior corona radiata	0.005	0.008	3.24	0.0026
Medial lemniscus	0.009	0.017	3.18	0.0032
**RD**				
Retrolenticular part of internal capsule	0.034	0.026	7.61	<0.0001
Fornix/stria terminalis	0.027	0.026	6.12	<0.0001
Cingulum-cingulate gyrus	0.025	0.026	5.76	<0.0001
Uncinate fasciculus	−0.040	0.044	5.34	<0.0001
Superior longitudinal fasciculus	0.009	0.012	4.78	<0.0001
Superior cerebellar peduncle	0.021	0.033	3.75	0.0007
Superior corona radiata	0.007	0.012	3.72	0.0007
Sagittal stratum	0.015	0.025	3.41	0.0017
**AD**				
Cingulum-cingulate gyrus	−0.025	0.012	12.11	<0.0001
Posterior corona radiata	0.015	0.013	6.49	<0.0001
Superior longitudinal fasciculus	0.012	0.011	6.29	<0.0001
Medial lemniscus	0.012	0.013	5.03	<0.0001
Anterior limb of internal capsule	0.008	0.010	4.45	0.0001
Posterior limb of internal capsule	0.007	0.010	4.16	0.0002
Posterior thalamic radiation	0.010	0.014	4.00	0.0003
Sagittal stratum	0.009	0.014	3.67	0.0008

*Group means and SDs of the symmetry index and the results of one-sample t-tests are shown. Statistical significance was determined by correction of α-levels for multiple comparison*.

Individual averages of symmetry index magnitudes were statistically larger in patients than controls in all DTI metrics but MD [FA: *t*(48) = 1.92, *p* = 0.030; MD: *t*(48) = 1.65, *p* = 0.052; RD: *t*(48) = 2.38, *p* = 0.011; AD: *t*(48) = 2.52, *p* = 0.007]. A separate examination, of within-individual across-ROI variations in symmetry index magnitudes, could not ascribe the larger asymmetry in the patient group to contributions by a select few patients with consistently extreme bilateral asymmetry. Thus, asymmetry in patients was in one way or another exaggerated beyond the asymmetry that was broadly observed in normal brains.

## Discussion

Using DTI metrics, we characterized bilateral white matter microstructural symmetry-asymmetry in normal individuals, and compared it to that of ED patients with a recent concussion. In normal subjects, the extent of symmetry varied among regions and individuals, and at least subtle forms of structural lateralization were common across regions. In patients, higher asymmetry was found overall as well as in a specific region, namely the corticospinal tract. Findings from two separate approaches, namely assessments of correlation and symmetry index, were in agreement. Results indicate that a concussion can manifest in brain asymmetry that deviates from a normal state.

Overall, values of FA tended to be higher, and MD, RD, and AD lower in our patient sample compared to controls, with a notable inconsistency in the middle cerebellar peduncle. FA values also tended to cluster more tightly in patients than controls, possibly indicating a ceiling effect. While the trend in FA may indicate an effect consistent with that reported for late acute injury (5–7 days post-concussion) (18), on account of our 2-week post-concussion recruitment window, our patient subjects likely represented a wide range of phases of response to the injury and changing diffusion characteristics (18, 19). Regardless of the MR scan timing, however, abnormality could be signaled as a deviation from normal benchmarks because the symmetry approach is based on intra-individual comparisons (5). Despite this benefit, the timing issue is still a weakness of this study. That is, although it is unlikely that the brain is affected evenly by the initial physical impact to the head, secondary effects involving commissural or bilateral changes (20) may alter symmetry-asymmetry characteristics over time. This possibility should be explored in a longitudinal study.

In this study, the corticospinal tract, identified at the medulla and the pons level in the atlas we used (15), stood out as a locus of asymmetry. Previously, we addressed whether anatomical findings after a concussive event could be biased in particular populations by examining patients with concussion from ED and athlete cohorts separately (8). The particularity of the corticospinal tract could be due to its vulnerability as well as variable sensitivity to concussion in general, but the mechanisms of injuries elucidating this particularity could not be identified due to the study design. Alternatively to the mechanistic explanation, it is possible that injury to the corticospinal tract leads to signs such as balance or coordination problems, for which a concussed patient may be directed to an ED. Such a cohort may have been well-represented in our ED patient sample. If so, other patient cohorts, such as athletes and soldiers, may be found with different or additional patterns of asymmetry from those reported here. Of note is a recent report on soldiers with a past concussion (5), which also identified the corticospinal tract as well as inferior longitudinal fasciculus (a tract not studied presently) as loci of increased asymmetry compared to military controls without a history of concussion. It is further conceivable that an individual in a typical head impact situation, unaccompanied by a person trained to recognize signs and symptoms of concussion, may not be seen by a medical professional immediately, until other symptoms or signs become a nuisance. Such type of patients may present still different patterns of brain asymmetry.

We defined the ROIs according to a standard white-matter atlas that is readily available (15). The use of this atlas allowed for objective quantification of white matter characteristics that could be compared across subjects, and the approach can be easily replicated in other laboratories. However, anatomical details that can be provided by the atlas are limited in terms of the number of the specified white matter structures and sensitivity to small, localized changes; therefore, asymmetry identified with the atlas may not be directly relevant to correlates of possible functional impacts of a concussion.

Lastly, the present findings cannot be generalized across ages because we selected adult subjects to control for potential developmental variables. Aging after maturity may also interact with the state of white matter symmetry following a concussion (5). Characterization of brain symmetry in normal development and aging would be an important avenue of research by itself as well as in defining abnormality.

To conclude, interpretation of brain asymmetry requires prudence. We showed that bilateral asymmetry reflecting disruption of a normal state can be found in patients after a concussion, but the study bore a number of further questions. The clinical utility of characterizing post-concussion pathology as abnormal brain asymmetry merits further exploration.

## Data Availability Statement

The raw data supporting the conclusions of this article will be made available by the authors, without undue reservation.

## Ethics Statement

The studies involving human participants were reviewed and approved by the Institutional Review Board of the Weill Cornell Medical College. The patients/participants provided their written informed consent to participate in this study.

## Author Contributions

JM, JG, and PM designed experiments and oversaw data collection. JMM, GS, and EP processed the imaging data. JM conducted the statistical analyses and drafted the manuscript. All authors contributed to the interpretation of data and to revising the work. All authors contributed to the article and approved the submitted version.

## Conflict of Interest

PM declares research support from GE Healthcare as well as service on the Medical Advisory Board of the GE-NFL Head Health Initiative. The remaining authors declare that the research was conducted in the absence of any commercial or financial relationships that could be construed as a potential conflict of interest.

## Abbreviations

ED: emergency department
DTI: diffusion tensor imaging
MR: magnetic resonance
AD: axial diffusivity
RD: radial diffusivity
MD: mean diffusivity
FA: fractional anisotropy
LOC: loss of consciousness
PTA: post-traumatic amnesia
ROI: region of interest
SD: standard deviation.
